# NSP2 Is Important for Highly Pathogenic Porcine Reproductive and Respiratory Syndrome Virus to Trigger High Fever-Related COX-2-PGE2 Pathway in Pigs

**DOI:** 10.3389/fimmu.2021.657071

**Published:** 2021-04-29

**Authors:** Li Du, Honglei Wang, Fang Liu, Zeyu Wei, Changjiang Weng, Jun Tang, Wen-hai Feng

**Affiliations:** ^1^ State Key Laboratory of Agrobiotechnology, College of Biological Sciences, China Agricultural University, Beijing, China; ^2^ Ministry of Agriculture Key Laboratory of Soil Microbiology, College of Biological Sciences, China Agricultural University, Beijing, China; ^3^ Department of Microbiology and Immunology, College of Biological Sciences, China Agricultural University, Beijing, China; ^4^ State Key Laboratory of Veterinary Biotechnology, Harbin Veterinary Research Institute of Chinese Academy of Agricultural Sciences, Harbin, China; ^5^ College of Veterinary Medicine, China Agricultural University, Beijing, China

**Keywords:** HP-PRRSV, NSP2, fever, PGE2, microglia

## Abstract

In 2006, atypical porcine reproductive and respiratory syndrome (PRRS) caused by a highly pathogenic PRRSV (HP-PRRSV) strain broke out in China. Atypical PRRS is characterized by extremely high fever and high mortality in pigs of all ages. Prostaglandin E2 (PGE2) derived from arachidonic acid through the activation of the rate-limiting enzyme cyclooxygenase type 1/2 (COX-1/2) plays an important role in fever. Here, we showed that HP-PRRSV infection increased PGE2 production in microglia *via* COX-2 up-regulation depending on the activation of MEK1-ERK1/2-C/EBPβ signaling pathways. Then, we screened HP-PRRSV proteins and demonstrated that HP-PRRSV nonstructural protein 2 (NSP2) activated MEK1-ERK1/2-C/EBPβ signaling pathways by interacting with 14-3-3ζ to promote COX-2 expression, leading to PGE2 production. Furthermore, we identified that the amino acid residues 500-596 and 658-777 in HP-PRRSV NSP2 were essential to up-regulate COX-2 expression and PGE2 production. Finally, we made mutant HP-PRRS viruses with the deletion of residues 500-596 and/or 658-777, and found out that these viruses had impaired ability to up-regulate COX-2 and PGE2 production *in vitro* and *in vivo*. Importantly, pigs infected with the mutant viruses had relieved fever, clinical symptoms, and mortality. These data might help us understand the molecular mechanisms underlying the high fever and provide clues for the development of HP-PRRSV attenuated vaccines.

## Introduction

Porcine reproductive and respiratory syndrome (PRRS) is one of the greatest threats to the swine industry worldwide. PRRS was first reported in America in 1987, which is characterized by respiratory tract distress in piglets and reproductive failures in pregnant sows (1). The causative agent, PRRS virus (PRRSV), was identified in Europe and America in 1991 and 1992, respectively. PRRSV is an enveloped and single-stranded positive-sense RNA virus, which belongs to the order *Nidovirales*, family *Arteriviridae*, and genus *Arterivirus* (2). Its genome contains at least 11 open reading frames (ORFs): ORF1a, ORF1b, ORF2a, ORF2b, ORFs 3 to 7, and the newly identified ORF5a and ORF2 (TF), of which ORF1a and ORF1b encode all the nonstructural proteins (NSPs) (3). In 2006, an unparalleled large-scale porcine disease caused by the highly pathogenic PRRSV (HP-PRRSV) strain with a discontinuous 30-amino-acid deletion in NSP2 protein broke out in China (4). HP-PRRSV-infected pigs have higher body temperatures, reaching 40-42°C, and the affected pigs of all ages show high morbidity and high mortality (4). Our previous report has indicated that the survival period of HP-PRRSV-infected pigs is negatively related to body temperatures (5).

Fever is the manifestation of an increase in body temperatures due to infections or inflammatory reactions (6). Studies have shown that prostaglandins play an indispensable role in the febrile response (7–9). Prostaglandins are members of the arachidonic acid family. Of them, prostaglandins E2 (PGE2) is the most abundant, which binds to four cognate G-protein-coupled receptor subtypes (EP1, EP2, EP3, and EP4) to regulate multiple responses to illnesses and psychological stresses (10). The synthesis of PGE2 is strictly controlled by sequential enzymatic processes, of which the rate-limiting enzyme cyclooxygenase type 1/2 (COX-1/2), also known as prostaglandin G/H synthase, plays a key role in the synthesis of PGE2 from arachidonic acid (11).

PGE2 is the common terminal mediator of pyrogens and functions in the hypothalamus frontal nucleus (12). Our previous study shows that PGE2 is induced in porcine alveolar macrophages (PAMs) by HP-PRRSV infection (5). The increase of peripheral PGE2 may be one of the causes of HP-PRRSV-induced high fever in pigs. However, over years, there is no direct evidence showing that peripheral PGE2 can travel or circumvent the blood-brain barrier to the brain to elicit fever (13). Studies have also shown that the peripheral PGE2 is eliminated across the blood-brain barrier in normal mice (14). Recently, more reports have supported the theory that brain-derived PGE2 is much more critical to induce fever. For example, Wilhelms and collaborators demonstrate that mice with selective deletion of COX-2 in the brain endothelial cells have attenuated fever response after injections with IL-1β and LPS (15). And, several studies have shown that COX-2 is abundantly expressed in brains (16–18). PRRSV is highly restricted to monocyte/macrophage lineage (19). Microglia are resident macrophages in the central nervous system (CNS) parenchyma and have been confirmed to support PRRSV replication *in vitro* (20). Thus, it is necessary to explore whether HP-PRRSV infection can enhance PGE2 production in microglia.

In this study, we demonstrated that NSP2 was important for HP-PRRSV to up-regulate COX-2 and PGE2 production in microglia. Deletion of residues 500-596 and 658-777 in HP-PRRSV NSP2 repressed its ability to induce COX-2 and PGE2 production. Importantly, compared to wild-type HP-PRRSV, the mutant HP-PRRS viruses with deletions of these critical residues in NSP2 had impaired ability to induce both COX-2 and PGE2 production. Finally, we demonstrated that fever response and mortality were relieved in pigs infected with the mutant HP-PRRS viruses. Our results may provide some new insights into the mechanisms about HP-PRRSV-triggered high fever response.

## Materials and Methods

### Cell Culture and Virus Preparation

HEK-293T, Hela, BV-2, and Marc-145 (PRRSV-permissive) cells were maintained in Dulbecco’s modified Eagle’s medium (DMEM) supplemented with 10% fetal bovine serum (FBS) and 1% penicillin-streptomycin. Porcine alveolar macrophages (PAMs) obtained by post-mortem lung lavage of 6-8 week-old specific-pathogen-free (SPF) pigs and 3D4/21 cells (a cell line derived from porcine alveolar macrophage) were cultured in RPMI 1640 supplemented with 10% FBS and 1% penicillin-streptomycin. Primary microglia were obtained as previously described with mild modifications (20, 21). Briefly, cerebral hemispheres obtained from post-mortem newborn pigs were washed three times with cold DMEM containing 2% penicillin-streptomycin. Meninges and blood vessels were then carefully removed. Next, the tissues were cut mechanically by scissors and treated with 0.25% trypsin without EDTA and DNase I (40 kunitz units/mL) for 30 min at 37°C. After several washes with DMEM-F12 supplemented with 10% FBS and 1% penicillin-streptomycin, cells were collected by centrifugation and cultured in DMEM-F12 supplemented with 10% FBS and 1% penicillin-streptomycin. The growth medium was replaced every 3-4 days until primary microglia were separated from mixed glia. Primary microglia were loosed by shaking at 180 rpm for 2 h at 37°C on an orbital shaker, and then harvested by centrifugation at 400 × g for 10 min. Microglia were cultured in DMEM-F12 with 10% FBS and 1% penicillin-streptomycin for further experiments.

Two PRRSV-2 strains were used in this study: CH-1a (a traditional PRRSV-2 strain isolated in China; GenBank accession, AY032626) and HV (a HP-PRRSV isolate; GenBank accession, JX317648). Viruses were propagated and titrated in Marc-145 cells or PAMs.

### Reagents and Antibodies

The COX-1 inhibitor (Sc-560) and an enzyme-linked immunosorbent assay (ELISA) kit for porcine PGE2 were purchased from Cayman systems. The COX-2 inhibitor (celecoxib) and the 14-3-3 inhibitor (BV02) were from Sigma. NF-κB inhibitor (BAY11-7082), p38MAPK inhibitor (SB203580), PKC inhibitor (GF-109203X), JNK inhibitor (SP600125) and MEK1 inhibitor (AZD6244) were from Selleck. All inhibitors were reconstituted in dimethyl sulfoxide (DMSO), and DMSO was used as the solvent control for all experiments involving treatment with inhibitors. TRIzol was from Invitrogen Life Technology. The Dual-Glo luciferase assay system was from Promega.

Antibodies against COX-1, ERK, p-ERK, p-C/EBPβ, and 14-3-3ζ were purchased from Cell Signaling Technology. Anti-COX-2 antibody was from Merk. And antibodies against β-actin and Flag were obtained from Sigma. Anti-HA antibody was from Santa Cruz, and anti-C/EBPβ antibody was purchased from GeneTex. Anti-NSP2 antibody was a gift from Dr. Chang-jiang Weng (Harbin Veterinary Research Institute, CAAS, China).

### Construction of Porcine COX-2 Promoters

Genomic DNA was extracted from PAMs and purified by using a DNA extraction kit (Axygen). The fragment of porcine COX-2 gene promoter flanking the 5’ COX-2 gene (NC_010451.4) was cloned by specific primers listed in **Table 1**. The obtained 1555-bp Sus scrofa COX-2 promoter sequence (nucleotides -1546 to +9) relative to the translation initiation site (+1) was sub-cloned into the luciferase (Luc) reporter vector pGL3-basic (-1546/+9-Luc). The mutants with truncated mutations of the COX-2 promoter were then constructed and inserted into the luciferase reporter vector pGL3-basic (-987/+9-Luc, -773/+9-Luc, -541/+9-Luc, -347/+9-Luc, -177/+9-Luc, and -77/+9-Luc). The truncated mutants of the COX-2 promoter were constructed using the primers listed in **Table 1**. The COX-2 promoter with deleted C/EBPβ element (-177/+9-(ΔC/EBPβ)-Luc) was generated by multiple rounds of PCR using -177/+9-Luc as a template, and the primers for C/EBPβ deletion in COX-2 promoter (-177/+9-Luc) were listed in **Table 1**. The mutated DNAs were then cloned into the pGL3-basic vector and verified by sequence analysis.

**Table 1:** Primers for truncated and deleted sequence of COX-2 promoter.

Primer name	Sequence
-1546/+9-Luc (sense)	GGGGTACCTGAAGCAGCAGAAGGGGGCAGTAA
-987/+9-Luc (sense)	GGGGTACCGGGCAGAAAAGACAAAAAGGCAAAC
-773/+9-Luc (sense)	GGGGTACCATACACAGTAAGTGATCACGATCAG
-541/+9-Luc (sense)	GGGGTACCAGGTCTGTCCAGACTGTGACTT
-347/+9-Luc (sense)	GGGGTACCCCCGAGGAAAGAAAGGAATC
-177/+9-Luc (sense)	GGGGTACCTATCTTACCCCCTCCTCCCA
-77/+9-Luc (sense)	GGGGTACCGTCACGTGGGCTTAGTTT
General antisense^a^	TCCCCCGGGTGTGACGCTTGCGGCAAGTTGA
-177/+9-(△C/EBPβ)-Luc (sense)	CCACTCCCGGTCTTATTTAAGCAG
-177/+9-(△C/EBPβ)-Luc (antisense)	CTGCTTAAATAAGACCGGGAGTGG

Nucleotide +1 represents the site of transcription initiation of the COX-2 promoter, and a truncated COX-2 promoter was cloned at the Kpn I and Sma I sites in the pGL3-basic luciferase reporter vector. Underlining shows the enzyme site. ^a^The antisense primers of -1546/+9-Luc, -987/+9-Luc, -773/+9-Luc, -541/+9-Luc, -347/+9-Luc, -177/+9-Luc, and -77/+9-Luc.

### Luciferase Reporter Assays

Marc-145 cells were prepared in a 24-well plate and co-transfected with a mixture of luciferase reporter plasmids and pRL-TK renilla luciferase plasmids using Lipofectamine LTX and Plus Reagent (Invitrogen). Twelve hours later, cells were infected with PRRSV at an MOI of 1. Cells were harvested at 24 hours post-infection, and luciferase activity analysis was performed according to the manufacturer’s instruction (Promega).

### Construction of PRRSV Protein Gene Expression Vectors and NSP2 Mutants

Genes encoding viral proteins were amplified from the HV genome and cloned into pcDNA3.1^+^. All constructed vectors were confirmed by sequencing (compared with the HP-PRRSV strain HV sequence). The HP-PRRSV NSP2 expression plasmid and NSP2 truncated mutants were constructed using specific primers shown in **Table 2**, and all of the truncated mutants’ products were sub-cloned into the pcDNA3.1^+^ vector. And the deletion mutants were constructed by Q5^®^ Site-Directed Mutagenesis Kit (NEB). The primers used in the construction of plasmids were listed in **Table 3**.

**Table 2:** Primers for truncated sequence of HP-PRRSV NSP2.

Primer name	Sequence
1-1196 (sense)	GGGGTACC *ATG*GCCGGAAAGAGAGCAAGGAAAAC
161-1196 (sense)	GGGGTACC *ATG*TCCGGATTTGATCCTGCCTGCCTTGACCGA
846-1196 (sense)	GGGGTACC *ATG*AGCTGCCAGGTTTTTAGCCTCGTTTCC
1031-1196 (sense)	GGGGTACC *ATG*TTCCCTTTTACACGTGCGACCAGG
322-1196 (sense)	GGGGTACC *ATG*TTGGGCAAGGACTCGGTCCCTCTGA
500-1196 (sense)	GGGGTACC *ATG*AGTGAGCCCGTACTTGTGC
597-1196 (sense)	GGGGTACC *ATG*GCGGGGGGGCAAGAAGTTGAGGAAGTCCT GAGTGAAAT
658-1196 (sense)	GGGGTACC *ATG*GAAGCATGCCTCAGCATCATGCGTGA
716-1196 (sense)	GGGGTACC *ATG*ATGATTCTCGAGACACCGCC
778-1196 (sense)	GGGGTACC *ATG*AAGGGAGAACCGGTCTGCGACCAACCT
1-1196 (antisense)^a^	GCTCTAGA *CTA*TCCCCCTGAAGGCTTGGAAATTTGCC

A truncated deletion of NSP2 was cloned at the KpnⅠ and SmaⅠ sites in the pcDNA3.1^+^ vector. Underlining shows the enzyme site. ^a^The antisense primer of 1-1196, 161-1196, 846-1196, 1031-1196, 322-1196, 500-1196, 597-1196, 658-1196, 716-1196, and 778-1196.

**Table 3:** Sequence of primers for Q5^®^ Site-Directed Mutagenesis Kit mediating NSP2 deletion mutant.

Primer name	Sequence
NSP2 (△161-845) (sense)	CTGAGCTGCCAGGTTTTTAGCC
NSP2 (△161-845) (antisense)	TTCGACCGCATCCGGGGA
NSP2 (△500-596) (sense)	GCGGGGGGGCAAGAAGTTGAGGAAGTCCTGAGTGAAAT
NSP2 (△500-596) (antisense)	CATAGGTGTCATCGGCTCGGATGGT
NSP2 (△597-657) (sense)	GAAGCATGCCTCAGCATC
NSP2 (△597-657) (antisense)	CTCCAGGATGCCCATGTT
NSP2 (△658-777) (sense)	AAGGGAGAACCGGTCTGCGACCAACCT
NSP2 (△658-777) (antisense)	TTTTTCCTTTTGGAGATGCCCACTGC

### RNA Isolation and Quantitative Real-Time PCR

RNA was extracted with TRIzol reagent (Invitrogen Life Technology) following the manufacturer’s instruction. RNA (1 μg) was then reverse-transcribed to cDNA using M-MLV reverse transcriptase according to the manufacturer’s instructions (Takara). Quantitative real-time PCR (qRT-PCR) analysis was performed using FastSYBR Mixture with ROX (Cwbiotech) on the ViiA™7 qRT-PCR System (Applied Biosystems). Gene-specific primers for GAPDH, COX-1, COX-2, and ORF7 were listed in **Table 4**.

**Table 4:** Quantitative RT-PCR primers.

Primer name	Sequence
sus scrofa-GAPDH (sense)	CCTTCCGTGTCCCTACTGCCAAC
sus scrofa -GAPDH (antisense)	GACGCCTGCTTCACCACCTTCT
sus scrofa-COX-2 (sense)	GTGGGGCATGAGGTCTTTGG
sus scrofa-COX-2 (antisense)	CAGCCTGCTCGTCTGGAACA
sus scrofa-COX-1 (sense)	CGAGAAGTGCCTCCCAAACTCC
sus scrofa-COX-1 (antisense)	AAGCCCATCTCGCCACCAAACG
human-GAPDH (sense)	CGGGGCTCTCCAGAACATC
human-GAPDH (antisense)	CTTCGACGCCTGCTTCAC
human-COX-2 (sense)	GCTGTTCCCACCCATGTCAA
human-COX-2 (antisense)	AAATTCCGGTGTTGAGCAGT
ORF7 (sense)	CCCGGGTTGAAAAGCCTCGTGT
ORF7 (antisense)	GGCTTCTCCGGGTTTTTCTTCCTA

### ELISA for PGE2

PGE2 in cerebrospinal fluid and cell culture supernatants was analyzed using an ELISA kit (Cayman Systems) according to the instruction manual.

### Inhibition of Signaling Transduction Pathways

Cells were pretreated with DMSO, PKC inhibitor GF-109203X (GF) (2 μM), p38MAPK inhibitor SB203580 (SB) (10 μM), MEK1 inhibitor AZD6244 (AZD) (10 μM), NF-κB inhibitor BAY11-7082 (BAY) (2 μM), or JNK inhibitor SP600125 (SP) (10 μM) for 1 hour, and then infected with or without HP-PRRSV at an MOI of 0.1 in the presence of inhibitors. Or cells were transfected with NSP2 plasmids using Lipofectamine 3000 Reagent, and then treated with DMSO and inhibitors at 6 hours post-transfection. At 6 hours post-infection or 18 hours after adding inhibitors, supernatants were harvested for the analysis of PGE2 by ELISA, and cells were harvested for COX-2 mRNA analysis by real-time PCR, protein detection using Western-blot and luciferase analysis.

### siRNA Knockdown

Cells were prepared in 12-well plates, and then small interfering RNA (siRNAs) oligonucleotides specific for the C/EBPβ, 14-3-3β, 14-3-3ζ, 14-3-3ϵ target genes, and a nonspecific control (NC) (Gene-Pharma) were transfected into cells using Hiperfect transfection reagents (Qiagen) or co-transfected with NSP2 using jetPRIME^®^ Transfection Reagent (Polyplus). At 24 hours post-transfection, cells were inoculated with PRRSV. Cells were harvested for real-time PCR or Western-blot analysis to evaluate the efficiency of knockdown and the expression of COX-2.

### Immunoprecipitation and Western-Blot

Immunoprecipitation and immunoblotting were performed as described previously. In brief, at 24 hours post-transfection, cell lysates were prepared in lysis buffer (50 mM Tris-Cl at pH8.0, 150 mM NaCl, 1% Triton X-100, 1 mM DTT, protease inhibitor cocktail and 10% glycerol), and were immunoprecipitated with M1 (SIGMA) for 4 hours at 4°C. Beads were washed and boiled. Protein samples were separated by SDS-PAGE, transferred to polyvinylidene diluoride (PVDF) membrane, and probed with antibody against HA to visualize the immunoprecipitated proteins.

### Rescue of PRRSV Mutants

The construction strategy was illustrated in **Figure 8A**. Briefly, the plasmid containing assembled genome of HV (pcDNA3.1-HV) constructed in our previous study (22) was cut with Spe I and Afl II to get 439-6478 bp of the HV deleted mutant pcDNA3.1-HV [pcDNA3.1-HV (Δ439-6478)]. Then, three overlapping fragments (F1, F2, and F3) spanning 439-6478 bp of HV were amplified using the specific primers listed in **Table 5**. The Q5^®^ Site-Directed Mutagenesis Kit was used to generate deleted or point mutations [F2 (Δ500-596), F2 (Δ658-777), and F2 (Δ500-596/658-777)]. Finally, using HiFi DNA Assembly Master Mix (NEB), pcDNA-HV-Δ500-596, pcDNA-HV-Δ658-777, and pcDNA-HV-Δ500-596/658-777 were constructed by linking the F1, F2, F3, and pcDNA-HV (Δ439-6478).

**Table 5:** Sequences of primers for recombined virus construction.

Primer name	Sequence
F1 fragment (sense)	CGATCTTTCCGATTGCACGAATGA
F1 fragment (antisense)	GCGTGTTTTCCTTGCTCTCTTTCCGGC
F2 fragment (sense)	GCCGGAAAGAGAGCAAGGAAAACACGC
F2 fragment (antisense)	CCGCGGCGACATCCGGGGATCTTTGGCAGGTTG
F3 fragment (sense)	CCAAAGATCCCCGGATGTCGCCGCGGGAGTC
F3 fragment (antisense)	GGGGCAGGAAGGCATAGGTGCTTAA

Underlining shows the overlapping sequence.

The constructed pcDNAs (2.5 μg/well) were transfected in HEK-293T cells using Lipofectamine^®^3000 Reagent when cultures were approximately 90% confluent in six-well plates. Supernatants were collected at 24 hours post-transfection and the rescued virus was designated P0. The P0 virus was then used to infect PAMs to generate P1 virus. Immunofluorescence assay was performed for virus detection. Then, P1 was serially passaged on PAMs to assess the stability of the rescued mutant PRRSVs. Rescued viruses were designated as Δ500-596, Δ658-777, and Δ56, respectively.

### Animal Experiment

Thirty four-week-old SPF piglets were randomly divided into five groups (six piglets per group). Piglets were intranasally inoculated with 2 mL (10^5^ TCID_50_ virus/mL) wt HP-PRRSV strain HV, Δ500-596, Δ658-777, and Δ56, respectively. Pigs in control group were intranasally inoculated with 2 mL medium. Three piglets from each group were euthanized at 6 days post-inoculation, while the rest were monitored daily for clinical signs. The rectal temperature of the piglets was measured every day until pigs died or euthanized. Plasma was collected at 0, 2, 4, 6, 10, 13, 16, 21, 24, and 28 days post-infection. Tissues and cerebrospinal fluid of piglets euthanized at 6 days post-inoculation or died later were collected. Tissues were fixed in 10% neutral-buffered formalin and routinely processed for histological examination, or snap-frozen in liquid nitrogen and stored at -80°C for RNA isolation (23). Cerebrospinal fluids were stored at -80°C for ELISA assay.

### Statistical Analysis

All the experiments were conducted with at least three independent replicates. Statistical analysis was performed using GraphPad Prism software, and differences in data were analyzed by Student’s *t*-test. Significance was allowed if the *P* value was less than 0.05. *, *P < 0.05*.

## Results

### HP-PRRSV Infection Up-Regulates COXs and PGE2 Production in Microglia

**Figure 1:**
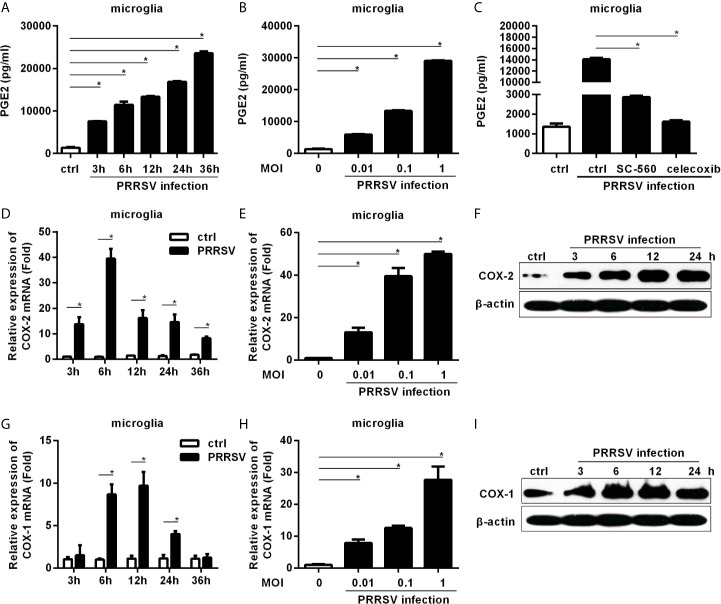
COXs and PGE2 are induced by HP-PRRSV infection in microglia. **(A)** Microglia were infected with medium alone, or HP-PRRSV strain HV (MOI, 0.1). Supernatants were harvested at 3, 6, 12, 24, and 36 hours post-infection, and PGE2 in supernatants (pg/mL) was determined by ELISA. **(B)** Microglia were infected with HV at an MOI of 0.01, 0.1, or 1. Supernatants were harvested at 6 hours post-infection, and PGE2 in supernatants (pg/mL) was determined by ELISA. **(C)** Microglia were pretreated with DMSO, COX-1 inhibitor sc-560 (1 μM), or COX-2 inhibitor celecoxib (1 μM) for 1 hour, and then mock-infected or infected with HV at an MOI of 0.1. At 12 hours post-infection, supernatants were harvested for PGE2 analysis using ELISA. **(D)** Microglia were inoculated with medium alone, or HP-PRRSV strain HV at an MOI of 0.1. COX-2 expression was analyzed using real-time PCR at 3, 6, 12, 24, and 36 hours post-inoculation. Results were normalized to GAPDH and expressed as fold induction over medium alone. **(E)** Microglia were infected with HP-PRRSV strain HV at an MOI of 0.01, 0.1, or 1, and total RNAs were extracted from cell lysates at 6 hours post-infection. COX-2 mRNA level was quantified by real-time PCR. **(F)** COX-2 protein levels in microglia infected with HP-PRRSV strain HV (MOI, 0.1) were analyzed using Western-blot at 3, 6, 12, and 24 hours post-inoculation. β-actin was set up as a loading control. **(G)** COX-1 mRNA level in microglia infected with HP-PRRSV strain HV (MOI, 0.1) was quantified by real-time PCR at 3, 6, 12, 24, and 36 hours post-infection. **(H)** HP-PRRSV strain HV infected microglia at an MOI of 0.01, 0.1, 1, and COX-1 mRNA level was quantified by real-time PCR at 12 hours post-infection. **(I)** COX-1 protein levels in microglia infected with HV (MOI, 0.1) were assessed by Western-blot at 3, 6, 12, and 24 hours post-infection. Data are representative of three independent experiments (mean ± SEM). Statistical analysis was performed by Student’s *t*-test. *, *P < 0.05*.

Since PRRSV infects microglia, we first investigated whether PRRSV infection up-regulated PGE2 production in microglia. As shown in **Figure 1A**, PGE2 production was significantly induced in microglia by HP-PRRSV infection, with 2.25-, 2.34-, 3.42-, 5.04-, and 7.05-fold increase at 3, 6, 12, 24, and 36 hours post-infection, respectively. The up-regulation of PGE2 production by HP-PRRSV was in a dose-dependent manner (**Figure 1B**). When we added COXs inhibitors, HP-PRRSV-induced PGE2 production was significantly inhibited (**Figure 1C**). Sc-560 (a inhibitor of COX-1) and celecoxib (a specific inhibitor of COX-2) suppressed PGE2 production with ~80% and 90% reductions, respectively, implying that PGE2 up-regulation in microglia by HP-PRRSV infection is dependent on the activation of COXs. We next explored the COXs expression in microglia infected with HP-PRRSV. Our results showed that COX-2 mRNA level was increased and peaked at 6 hours post-infection (about 39-fold induction) (**Figures 1D, E**). To further confirm COX-2 expression in microglia, we examined COX-2 protein levels by Western-blot. As shown in **Figure 1F**, COX-2 was gradually up-regulated in microglia by HP-PRRSV infection. Similarly, COX-1 mRNA (about 9.7-fold induction at 12 hours post-infection) and protein levels were also increased in microglia infected with HP-PRRSV (**Figures 1G–I**). These data suggest that HP-PRRSV infection up-regulates COXs production, subsequently leading to enhanced PEG2 production. COX-2 is an inducible isoform, whereas COX-1 is constitutively expressed in most tissues (11). Moreover, COX-2 has been considered as the main enzyme regulating PGE2 production in fever responses (24). And it is also reported that sc-560 can inhibit PGE2 synthesis independent of COX-1 (25). Therefore, we next focused on how HP-PRRSV up-regulated COX-2.

### The MEK1-ERK1/2 Pathways Are Required for PRRSV-Induced COX-2 and PGE2 Production in Microglia

**Figure 2:**
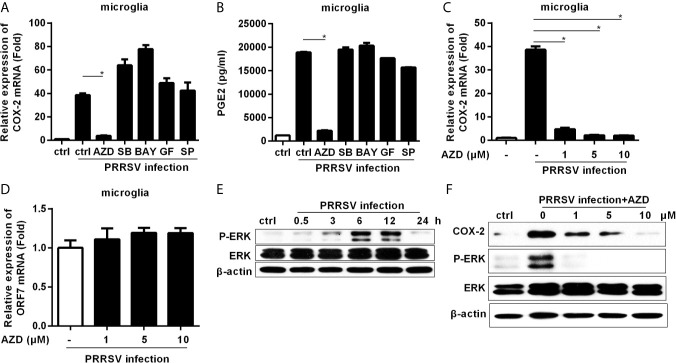
Highly specific MEK1 inhibitor impaired HP-PRRSV-induced COX-2 and PGE2 production in microglia. **(A, B)** Microglia were pretreated with DMSO control or inhibitors of MEK1 (AZD6244 [AZD]), p38MAPK (SB203580 [SB]), NF-κB (BAY11-7082 [BAY]), PKC (GF109203X [GF]), and JNK MAPK (SP600125 [SP]) for 1 hour, and then cells were mock infected or infected with HP-PRRSV strain HV (MOI, 0.1). At 6 hours later, cells were harvested for COX-2 mRNA analysis by real-time PCR **(A)**, and cell culture supernatants were harvested for testing PGE2 production by ELISA **(B)**. **(C, D)** Microglia were pretreated with DMSO or AZD at the indicated concentrations for 1 hour, and then mock infected or infected with HP-PRRSV strain HV at an MOI of 0.1. At 6 hours post-infection, cells were harvested for COX-2 **(C)** and ORF7 **(D)** analysis using real-time PCR. **(E)** Microglia were inoculated with HP-PRRSV strain HV (MOI, 0.1), and cells were harvested at 0.5, 3, 6, 12, and 24 hours post-infection. ERK1/2 and p-ERK1/2 were determined by immunoblotting with β-actin as a reference control. **(F)** Microglia were pretreated with DMSO or AZD at the indicated concentrations for 1 hour, and then mock infected or infected with HP-PRRSV strain HV (MOI, 0.1). At 12 hours post-infection, COX-2, p-ERK1/2 and ERK1/2 were examined by Western-blot with β-actin as a reference control. Data are representative of three independent experiments (mean ± SEM). Statistical analysis was performed by Student’s *t*-test. *, *P < 0.05*.

To dissect the signaling pathways involved in COX-2 induction, microglia were pretreated with inhibitors of the key signaling molecules, including MEK1, p38 MAPK, NF-κB, PKC, and JNK, and then infected with HP-PRRSV at an MOI of 0.1. Our results showed that the highly selective MEK1 inhibitor (AZD) significantly impaired HP-PRRSV-induced COX-2 production in microglia (**Figure 2A**). Correspondingly, the enhancement of PGE2 by HP-PRRSV was also suppressed by the MEK1 specific inhibitor (**Figure 2B**). To further confirm the role of MEK1, microglia were pretreated with different concentrations of the highly selective MEK1 inhibitor before HP-PRRSV infection. As shown in **Figure 2C**, COX-2 expression was significantly suppressed by MEK1 inhibitor in a dose-dependent manner. To exclude the possible effects of MEK1 inhibitor on PRRSV replication, we analyzed PRRSV ORF7 expression. Our results showed that the highly selective MEK1 inhibitor at the used concentrations had no obvious effects on HP-PRRSV replication (**Figure 2D**). These data imply that MEK1 signaling pathway participates in HP-PRRSV-induced COX-2 production. ERK1/2 has been known as the downstream effector of Raf-MEK1. Thus, we next clarified whether ERK1/2 played a role in COX-2 up-regulation by HP-PRRSV infection. As expected, ERK1/2 phosphorylation was increased in microglia infected with HP-PRRSV (**Figure 2E**). MEK1 inhibitor also significantly inhibited HP-PRRSV-induced ERK1/2 activation, which was in accordance with the decrease of COX-2 expression (**Figure 2F**). Therefore, we conclude that HP-PRRSV infection induces COX-2 production in microglia through MEK1-ERK1/2 signaling pathways.

### C/EBPβ Is the Key Transcription Factor for HP-PRRSV-Induced COX-2 Production

**Figure 3:**
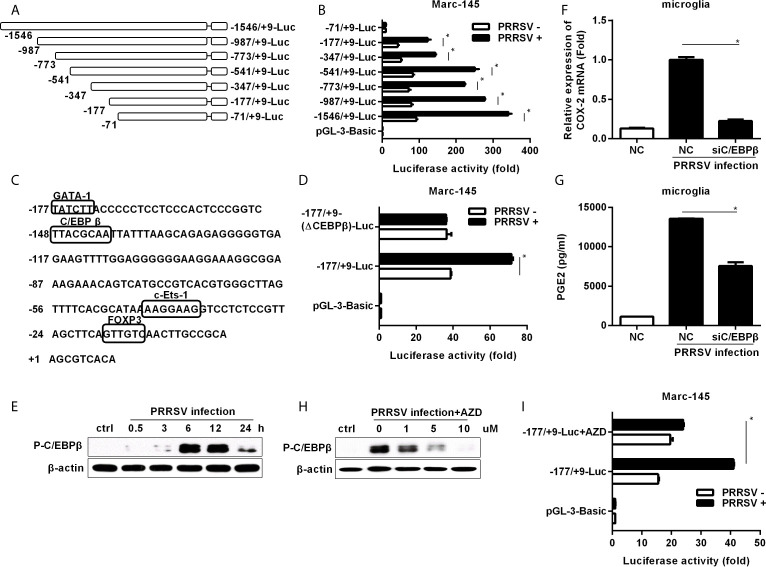
Cloning, sequence analysis, and characterization of porcine COX-2 promoter. **(A)** Schematic representation of the porcine COX-2 promoter and promoter deletion mutants inserted into pGL3-basic luciferase vectors: -1546/+9-Luc, -987/+9-Luc, -773/+9-Luc, -541/+9-Luc, -347/+9-Luc, -177/+9-Luc and -71/+9-Luc porcine COX-2 promoter vectors. The relative lengths and positions of the 5’ ends of these fragments are indicated. **(B)** The luciferase reporter vectors or pGL3-basic empty and pRL-TK renilla luciferase plasmids were co-transfected into Marc-145 cells. At 12 hours later, cells were mock infected or infected with PRRSV (MOI, 1), and then cells were harvested to determine luciferase activity at 24 hours post-infection. **(C)** Sequence analysis of the 186-bp porcine COX-2 gene 5’ flanking regions. The putative transcription factor binding sites were shown in boxes. **(D)** Marc-145 cells were transfected with the constructed mutant promoter for 12 hours, and then cells were mock infected or infected with PRRSV (MOI, 1). At 24 hours post-infection, cells were harvested for luciferase activity analysis. **(E)** Microglia were infected with HP-PRRSV strain HV (MOI, 0.1), and cells were harvested for p-C/EBPβ analysis at 0.5, 3, 6, 12, and 24 hours post-infection. **(F, G)** Microglia were transfected with siRNA targeting C/EBPβ for 24 hours, and then mock infected or infected with HP-PRRSV strain HV (MOI, 0.1). At 12 hours post-infection, cells were harvested for COX-2 mRNA analysis by real-time PCR **(F)**, and cell supernatants were collected for PGE2 production by ELISA **(G)**. **(H)** Cells were pretreated with the DMSO or AZD at the indicated concentrations for 1 hour, and then mock infected or infected with HP-PRRSV (MOI, 0.1). At 12 hours post-infection, cells were harvested for p-C/EBPβ analysis by Western-blot using a specific antibody against p-C/EBPβ. **(I)** Marc-145 cells were co-transfected with -177/+9-Luc porcine COX-2 promoter vector or the empty vector pGL3-basic and pRL-TK renilla luciferase plasmids. At 6 hours after transfection, cells were treated with DMSO or AZD (10 μM) for 1 hour, and then inoculated with PRRSV at an MOI of 1. At 24 hours post-infection, cells were lysed for luciferase activity analysis. Data are representative of three independent experiments (mean ± SEM). Statistical analysis was performed by Student’s *t*-test. *, *P < 0.05*.

To investigate the essential transcription factors involved in HP-PRRSV-mediated COX-2 expression, we cloned a 1555-bp fragment of the 5’ flanking region of the porcine COX-2 gene and constructed serial truncated COX-2 promoter fragment reporter plasmids (**Figure 3A**). Then, we assessed COX-2 promoter activities using luciferase assays. Our results showed that most of the constructed promoter activities were significantly elevated in HP-PRRSV-infected cells. However, the -71/+9-Luc promoter was not activated by HP-PRRSV infection (**Figure 3B**), suggesting that there might be some key regulatory elements in the region of -177 to +9 in COX-2 promoter. Using the bioinformatics analysis (http://alggen.lsi.upc.es and http://jaspar.genereg.net/), we found that there were several putative transcriptional regulatory elements (GATA-1, C/EBPβ, c-Ets-1, and FOXP3) in this region (**Figure 3C**). Studies have shown that COX-2 expression is regulated by C/EBPβ in many cell types and stimulation paradigms (26, 27). To evaluate the role of C/EBPβ in COX-2 induction by PRRSV infection, we generated COX-2 luciferase reporter (-177/+9-(ΔC/EBPβ)-Luc) with mutations in the C/EBPβ binding site. As expected, C/EBPβ binding site deletion significantly impaired COX-2 promoter activation by HP-PRRSV infection (**Figure 3D**), suggesting that the putative C/EBPβ motif might be the critical responsive element in porcine COX-2 promoter for COX-2 activation by HP-PRRSV infection.

To further confirm the role of C/EBPβ, we investigated whether C/EBPβ was activated in microglia infected with HP-PRRSV. As shown in **Figure 3E**, C/EBPβ was significantly activated. Next, we transfected microglia with specific siRNAs targeting C/EBPβ before HP-PRRSV infection. Our data showed that knockdown of C/EBPβ remarkably decreased COX-2 expression (**Figure 3F**) and PGE2 secretion (**Figure 3G**), suggesting that C/EBPβ is the key transcription factor for COX-2 and PGE2 production in microglia induced by HP-PRRSV infection. To investigate the relationship between MEK1-ERK1/2 and C/EBPβ signal transduction pathways in HP-PRRSV-induced COX-2 expression, we pretreated microglia with the highly specific MEK1 inhibitor. The results showed that C/EBPβ activation in response to HP-PRRSV infection was suppressed by MEK1 inhibitor (**Figure 3H**). And accordingly, the activation of COX-2 promoter (-177/+9) by HP-PRRSV was also inhibited by MEK1 inhibitor (**Figure 3I**). Collectively, these data indicate that C/EBPβ is the key transcription factor for COX-2 production in microglia infected with HP-PRRSV.

### HP-PRRSV NSP2 Induces COX-2 Production

**Figure 4:**
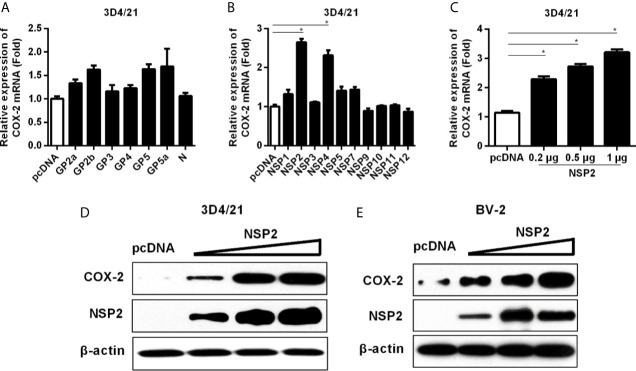
Determining the effect of HP-PRRSV NSP2 on COX-2 activation. **(A, B)** 3D4/21 cells were transfected with HP-PRRSV structural **(A)** and nonstructural protein **(B)** expression vectors. At 24 hours later, cells were harvested for COX-2 mRNA analysis by real-time PCR. **(C, D)** 3D4/21 cells were transfected with NSP2 expression plasmids at different doses (0.2, 0.5, 1 μg). At 24 hours later, total RNA was extracted from cell lysates. COX-2 mRNA was quantified by real-time PCR **(C)**, and cell lysates were harvested for COX-2 protein analysis by Western-blot **(D)**. **(E)** BV-2 cells were transfected with NSP2 expression plasmids at different doses (0.2, 0.5, 1 μg). At 24 hours later, COX-2 protein level was analyzed by Western-blot. Data are representative of three independent experiments (mean ± SEM). Statistical analysis was performed by Student’s *t*-test. *, *P < 0.05*.

To figure out which HP-PRRSV protein(s) up-regulates COX-2 production, we transfected with each of the structural and nonstructural genes (derived from the viral genome of HP-PRRSV HV strain) into 3D4/21 cells. As shown in **Figures 4A, B**, COX-2 was significantly induced by NSP2 and NSP4. Since NSP2 was the most efficient PRRSV protein to induce COX-2 production, we next focused on NSP2. To further confirm the ability of NSP2 to induce COX-2, we transfected 3D4/21 cells with different amounts of NSP2. Our results showed that COX-2 expression was increased by about 2.3, 2.7, 3.3-folds compared to the control vector when cells were transfected with 0.2, 0.5, and 1 μg of NSP2, respectively (**Figure 4C**). COX-2 protein level in 3D4/21 cells (**Figure 4D**) and BV-2 cells (a mouse microglia cell line) (**Figure 4E**) was also up-regulated by NSP2. These results indicate that HP-PRRSV NSP2 can induce COX-2 production.

### HP-PRRSV NSP2 Up-Regulates COX-2 Through MEK1-ERK1/2-C/EBPβ Pathways

**Figure 5:**
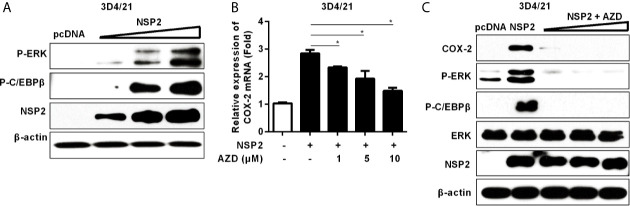
Analysis of the role of NSP2 in activating MEK1-ERK1/2-C/EBPβ pathway. **(A)** 3D4/21 cells were transfected with different amounts of NSP2 protein expression vector. At 24 hours post-transfection, harvested cells were lysed for Western-blot to examine levels of p-ERK, p-C/EBPβ, and NSP2. **(B, C)** 3D4/21 cells were transfected with NSP2 (0.5 μg). At 6 hours after transfection, cells were treated with DMSO or indicated concentrations of AZD. At 18 hours post-inoculation, cells were collected and subjected to the real-time PCR to measure COX-2 mRNA level **(B)** and Western-blot to detect COX-2, p-ERK, p-C/EBPβ, ERK, and NSP2 protein levels **(C)**. Data are representative of three independent experiments (mean ± SEM). Statistical analysis was performed by Student’s *t*-test. *, *P < 0.05*.

To investigate whether NSP2-induced COX-2 production is also through the activation of MEK1-ERK1/2-C/EBPβ pathway, we first analyzed the relative phosphorylation levels of ERK and C/EBPβ in NSP2-transfected 3D4/21 cells by Western-blot. A dose-dependent increase in the phosphorylation levels of ERK and C/EBPβ was observed (**Figure 5A**). In addition, NSP2-induced COX-2 expression was repressed by the highly specific MEK1 inhibitor in a dose-dependent manner (**Figure 5B**). Similarly, MEK1 inhibitor significantly suppressed NSP2-enhanced COX-2 production and ERK1/2 and C/EBPβ activations (**Figure 5C**). Taken together, we conclude that HP-PRRSV NSP2-mediated COX-2 production is regulated by MEK1-ERK1/2-C/EBPβ signaling pathways.

### HP-PRRSV NSP2 Activates MEK1-ERK1/2 Pathways by Interacting With 14-3-3ζ

**Figure 6:**
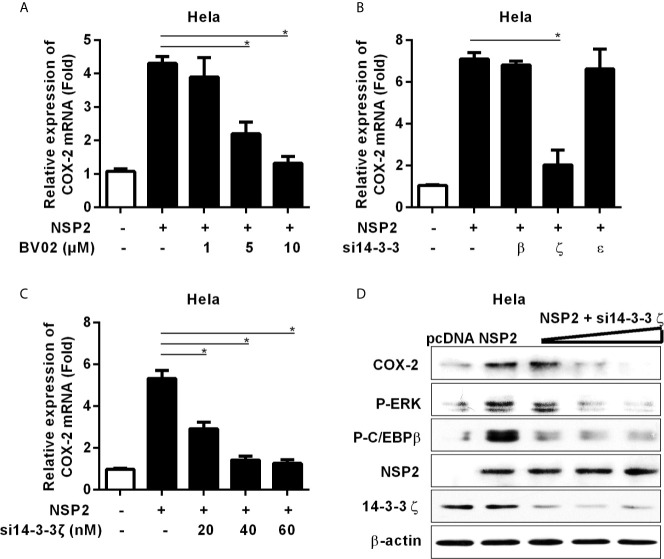
14-3-3ζ participates in NSP-2-induced COX-2 production. **(A)** Hela cells were transfected with NSP2 (0.5 μg). At 6 hours post transfection, cells were treated with DMSO or the pan-inhibitor of 14-3-3 protein family (BV02) at the indicated concentrations. At 18 hours post-inoculation, cells were collected and subjected to the real-time PCR to measure COX-2 mRNA level. **(B)** Hela cells were co-transfected with NSP2 and siRNA targeting 14-3-3β, 14-3-3ϵ, and 14-3-3ζ, respectively. At 24 hours later, cells were harvested for COX-2 mRNA analysis by real-time PCR. **(C, D)** Hela cells were co-transfected with NSP2 and siRNA targeting 14-3-3ζ at the indicated doses, respectively. Cells were harvested for COX-2 mRNA analysis by real-time PCR **(C)** and COX-2, p-ERK, p-C/EBPβ, 14-3-3ζ, and NSP2 protein analysis by Western-blot **(D)**. Data are representative of three independent experiments (mean ± SEM). Statistical analysis was performed by Student’s *t*-test. *, *P < 0.05*.

It has been reported that NSP2 interacts with 14-3-3 protein family, which play roles in critical regulatory processes in cells (28). To investigate whether NSP2 activates MEK1-ERK1/2 signal transduction by interacting with 14-3-3 proteins, we treated Hela cells with the pan-inhibitor (BV02) of 14-3-3 protein family before NSP2 transfection. Our data showed that BV02 significantly inhibited COX-2 production induced by NSP2 (**Figure 6A**), suggesting that 14-3-3 proteins are involved in NSP2-induced COX-2 production. To explore which specific 14-3-3 isoform (14-3-3β, γ, ϵ, ζ, or η) is essential for NSP2 to induce COX-2 expression, we co-transfected NSP2 and siRNAs targeting specific 14-3-3 isoforms into Hela cells. Our results showed that 14-3-3ζ-specific siRNAs remarkably impaired the ability of NSP2 to induce COX-2 expression (**Figures 6B, C**). In addition, knock-down of 14-3-3ζ suppressed MEK1-ERK1/2-C/EBPβ activation by NSP2 (**Figure 6D**). These data suggest that NSP2 activates MEK1-ERK1/2-C/EBPβ *via* interacting with 14-3-3ζ, subsequently leading to COX-2 activation.

### 500-596 and 658-777 aa Regions in the HV Domain Are Important for NSP2 to Activate COX-2 Induction

**Figure 7:**
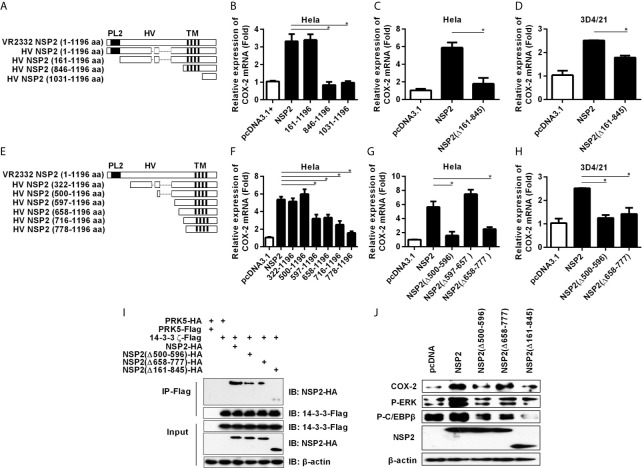
Analysis of functional domain of NSP2 for COX-2 up-regulation. **(A)** Schematic representation of various truncated mutants of HP-PRRSV strain HV NSP2. The dashed lines indicated the sequence naturally deleted as compared with the North American genotype representative virus VR-2332, including residue 481 and residues 532-560. PL2, the putative enzyme domain; HV, the hypervariable region; TM, the transmembrane region. **(B–D)** Hela cells **(B, C)** and 3D4/21 cells **(D)** were transfected with NSP2, NSP2 deletion mutant vectors, or pcDNA3.1^+^ empty vector. At 24 hours later, cells were harvested for COX-2 mRNA analysis by real-time PCR. **(E)** Schematic representation of various truncated mutants of HP-PRRSV strain HV NSP2. The dashed lines indicated the sequence naturally deleted as compared with the North American genotype representative virus VR-2332, including residue 481 and residues 532-560. **(F–H)** Hela cells **(F, G)** and 3D4/21 cells **(H)** were transfected with NSP2, NSP2 deletion mutant vectors, or pcDNA3.1^+^ empty vector. At 24 hours later, cells were harvested for COX-2 mRNA analysis by real-time PCR. **(I)** HEK-293T cells were co-transfected with Flag-tagged 14-3-3ζ and HA-tagged NSP2 deletion mutant expression plasmids. At 24 hours later, cells were harvested for immunoprecipitation. The expression of HA-tagged NSP2 and Flag-tagged 14-3-3ζ were analyzed by Western-blot. **(J)** Hela cells were transfected with NSP2 and NSP2 deletion mutants. At 24 hours later, cells were harvested to detect COX-2, p-ERK, p-C/EBPβ, and NSP2 protein levels by Western-blot. Data are representative of three independent experiments (mean ± SEM). Statistical analysis was performed by Student’s *t*-test. *, *P < 0.05*.

The structure of NSP2 contains four domains, including a papain-like protease 2 (PL2) domain (aa 1-160), a central hypervariable (HV) domain (aa 161-846), a transmembrane (TM) domain (aa 846-1031), and a tail domain (aa 1032-1196) (**Figure 7A**) (29–32). To determine which domain(s) of HP-PRRSV NSP2 is essential for COX-2 up-regulation, we constructed NSP2 mutants with amino-acid terminal deletions (**Figure 7A**), and then transfected them into Hela and 3D4/21 cells, respectively. Our results showed that deletion of 1-160 aa in NSP2 (NSP2 mutant: 161-1196) containing PL2 core domain did not affect its ability to enhance COX-2 expression (**Figure 7B**). However, the mutant without PL2 and HV domains (NSP2 mutant: 846-1196) and mutant without PL2, HV, and TM domains (NSP2 mutant: 1031-1196) had impaired ability to induce COX-2 production. Further analysis showed that deletion of HV domain [NSP2 (Δ161-845)] abrogated its ability to induce COX-2 production in Hela cells (**Figure 7C**) and 3D4/21 cells (**Figure 7D**). These data suggest that HV domain is essential for NSP2 to up-regulate COX-2 production.

To verify the amino acid sequences in NSP2 HV domain that are critical for NSP2 to up-regulate COX-2, we performed more systemic deletions in the HV domain (**Figure 7E**). We found that deletion of 499 amino acids from amino-acid terminal of NSP2 (NSP2 mutant: 500-1196 aa) did not affect its ability to induce COX-2 expression. However, further deletion of 777 amino acids (NSP2 mutant: 778-1196 aa) in NSP2 affected its ability to induce COX-2 expression, suggesting that residues 500-778 in NSP2 are essential for COX-2 up-regulation (**Figure 7F**). More detailed deletion mutants (NSP2 mutant: 597-1196 aa; NSP2 mutant: 658-1196 aa; NSP2 mutant: 716-1196 aa) also had impaired ability to induce COX-2 expression, implying that there might exist more than one functional region important for NSP2 to up-regulate COX-2 in the sequence 500-778 aa. Next, we generated NSP2 mutants without 500-596 aa [NSP2 (Δ500-596)], 597-657 aa [NSP2 (Δ597-657)], or 658-777 aa [NSP2 (Δ658-777)], and analyzed their abilities to induce COX-2 expression. As shown in **Figures 7G, H**, mutants NSP2 (Δ500-596) and NSP2 (Δ658-777) had impaired ability to activate COX-2, while NSP2 (Δ597-657) still efficiently induced COX-2 expression, suggesting that 500-596 aa and 658-777 aa regions are important for NSP2 to induce COX-2 production. To further confirm these results, we co-transfected Flag-tagged 14-3-3ζ with HA-tagged NSP2 or NSP2 mutants [including NSP2 (Δ500-596), NSP2 (Δ658-777), and NSP2 (Δ161-845)] into HEK-293T cells, and then used immunoprecipitation test to investigate the interaction between 14-3-3ζ and NSP2 or NSP2 mutants. Our results showed that less NSP2 mutant was co-immunoprecipitated with Flag-tagged 14-3-3ζ when compared with intact NSP2, indicating that deletion of 500-596 aa, 658-777 aa, or 161-845 aa in NSP2 HV domain affects the interaction between 14-3-3ζ and NSP2 (**Figure 7I**). Furthermore, we detected the protein level of COX-2 and C/EBPβ phosphorylation in cells transfected with intact NSP2 or its mutants: NSP2 (Δ500-596), NSP2 (Δ658-777), and NSP2 (Δ161-845). The results showed that these mutants were unable to efficiently activate ERK1/2 and C/EBPβ to induce COX-2 expression (**Figure 7J**).

Taken together, these data imply that the regions of 500-596 aa and 658-777 aa in the HV domain are critical for NSP2 to up-regulate COX-2.

### Recombinant HP-PRRSV With Mutated NSP2 Has Impaired Ability to Up-Regulate PGE2 Production *In Vitro*

**Figure 8:**
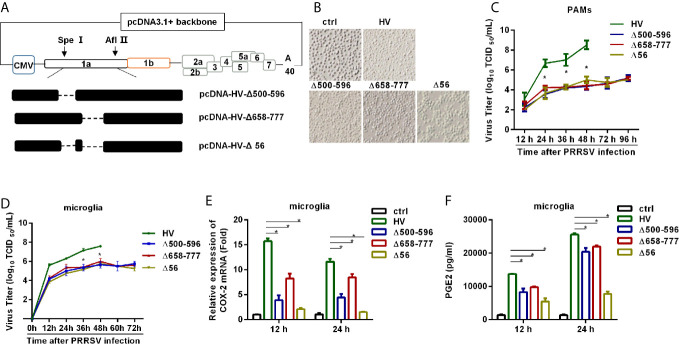
HP-PRRSV with deletion mutant NSP2 impairs PGE2 production. **(A)** Strategy for the construction of the recombinant cDNA clone. The plasmid containing assembled genome of HV (pcDNA3.1-HV) was treated with Spe I and Afl II to get 439-6478 bp of the HV deleted mutant pcDNA3.1-HV [pcDNA3.1-HV (Δ439-6478)], and then the Q5^®^ Site-Directed Mutagenesis Kit was used to generated deleted mutations. Finally, using HiFi DNA Assembly Master Mix, pcDNA-HV-Δ500-596 [Δ500-596], pcDNA-HV-Δ658-777 [Δ658-777], and pcDNA-HV-Δ500-596/658-777 [Δ56]) were constructed. **(B)** Representative images of cytopathic effects induced by wt HP-PRRSV strain HV and the mutant viruses at 48 hours post-infection. **(C, D)** Supernatants were collected at indicated times post virus infection, and titrated for viruses using TCID_50_ method. **(E, F)** Microglia were infected with HP-PRRSV strain HV and the mutant viruses at an MOI of 0.1. COX-2 expression was analyzed by real-time PCR **(E)** and PGE2 production was measured by ELISA **(F)** at 12 and 24 hours post-infection. Data are representative of three independent experiments (mean ± SEM). Statistical analysis was performed by Student’s *t*-test. *, *P < 0.05*.

Next, we tried to elucidate the role of NSP2 in HP-PRRSV-induced COX-2 expression and PGE2 production in the context of virus infection. We first constructed the mutant viruses either with the deletion of residues 500-596 aa, 658-777 aa, or both 500-596 aa and 658-777 aa in NSP2 based on the parental virus HP-PRRSV HV strain, and assigned the rescued viruses as Δ500-596, Δ658-777, and Δ56, respectively (**Figure 8A**). Then, we characterized the mutant viruses. As shown in **Figure 8B**, there were hardly any living PAMs remained at 48 hours post-infection with wild-type (wt) HP-PRRSV HV strain (MOI, 0.1). However, there were no obvious cytopathic effects observed in PAMs infected with Δ500-596, Δ658-777, or Δ56 (**Figure 8B**). The viral growth analysis showed that the recombined viruses grew much slower than the wild-type HV in PAMs (**Figure 8C**) and microglia (**Figure 8D**).

To determine whether deletion of residues 500-596 aa, 658-777 aa, or both 500-596 aa and 658-777 aa in NSP2 has effects on COX-2 expression and PGE2 secretion, we infected microglia with the mutant viruses Δ500-596, Δ658-777, Δ56, and wt HP-PRRSV HV, respectively. And then, we analyzed COX-2 expression and PGE2 secretion. Our results showed that the mutant virus Δ500-596, Δ658-777, or Δ56 inefficiently induced COX-2 expression (**Figure 8E**) and PGE2 production (**Figure 8F**) compared to the parental virus.

In summary, these findings imply that NSP2 is important for HP-PRRSV to induce COX-2 and PGE2 production, and the regions of 500-596 aa and 658-777 aa in NSP2 are the essential motifs.

### Mutant HP-PRRS Viruses Cannot Induce Higher Fever and Have Attenuated Pathogenicity *In Vivo*

Finally, we verified the role of NSP2 in HP-PRRSV-triggered high fever and evaluated the pathogenicity of the recombined viruses *in vivo*. We intranasally inoculated 6 4-week-old piglets in each group with medium, wt HP-PRRSV strain HV, Δ500-596, Δ658-777, or Δ56 at a dose of 2 mL x 10^5^/TCID_50_. At 6 days post-inoculation, 3 piglets in each group were euthanized to assess histopathologic lesions and PGE2 levels in cerebrospinal fluids, and the other 3 piglets in each group were continuously observed for disease symptoms for 28 days. Our results showed that piglets inoculated with wt HP-PRRSV HV strain had dyspnea, anorexia, lethargy, depression, and shivering until they died. One piglet in Δ500-596-inoculated group had dyspnea and died, and the other two only presented mild signs. Interestingly, the piglets infected with Δ658-777 or Δ56 had no obvious clinical symptoms and remained healthy until the end of experiment. No clinical signs were observed in the medium-infected group during the experiment period.

**Figure 9:**
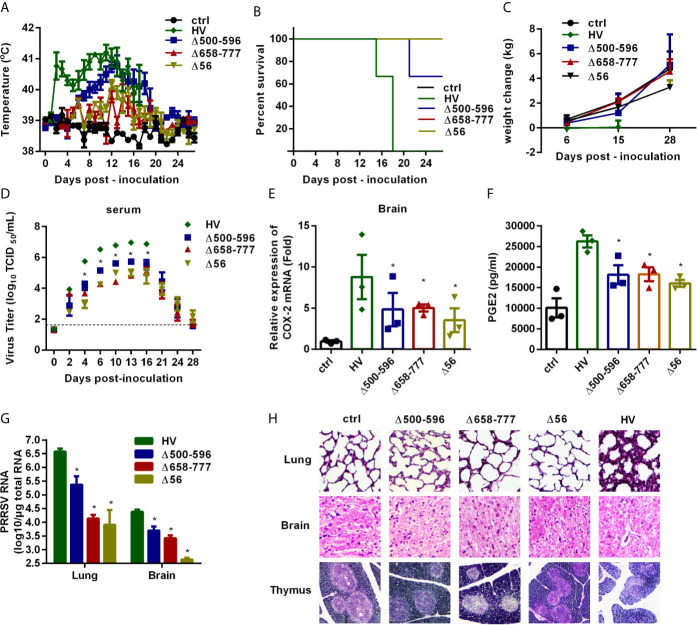
The mutant viruses Δ500-596, Δ658-777, and Δ56 are less virulent *in vivo*. Pigs were intranasally inoculated with HP-PRRSV strain HV, Δ500-596, Δ658-777, and Δ56 at a dose of 2 mL × 10^5^ TCID_50_ virus/mL, respectively. **(A)** Rectal temperature of piglets was measured every day post-infection, and shown as the means ± SEM, except the number of survival piglets in each group was less than two. **(B)** Survival curves of piglets in each group. **(C)** Weight changes of piglets in each group. **(D)** Virus titers in serum of piglets inoculated with the HV or mutant viruses were determined by real-time PCR, and known amounts of serially diluted *in vitro* transcript virus cDNA were used to generate a standard curve for each plate run. The limit of serum virus titers detection was indicated in dashed line. **(E–H)** Three of each group piglets were euthanized at 6 days post-inoculation, and tissues and cerebrospinal fluid of piglets were collected; COX-2 expression in brain was analyzed by real-time PCR **(E)**, and PGE2 production in cerebrospinal fluid of piglets was measured by ELISA **(F)**; virus RNA loads in tissues (lung and brain) were detected by real-time PCR **(G)**; tissues were fixed in 10% neutral-buffered formalin and routinely processed for histological examination, lung 10 × 10, brain 10 × 10, thymus 4 × 10 **(H)**. Statistical analysis was performed by Student’s *t*-test. *, *P < 0.05*.

High rectal temperature (above 40°C) was observed in pigs infected with wt HP-PRRSV HV strain as early as 2 days post-infection (**Figure 9A**). The average rectal temperature remained above 40°C from then and peaked around 10-14 days post-inoculation. The piglets inoculated with Δ500-596 exhibited moderate-higher rectal temperature started at day 6 post-inoculation, and then transiently reached a peak (~ 41°C) around 12 days post-infection. Nevertheless, piglets infected with Δ500-596 still had lower temperatures than the wt HP-PRRSV-infected piglets. However, piglets infected with Δ658-777 or Δ56 mutant virus exhibited significantly lower temperatures than piglets infected with HP-PRRSV. These piglets had temperatures similar to piglets inoculated with medium, except for small fluctuations from 12 to 15 days post-inoculation.

We also recorded the mortalities and weight changes of the inoculated animals. As shown in **Figure 9B**, all piglets in HV-inoculated groups died within 15-18 days post-infection, and one piglet inoculated with Δ500-596 died. Surprisingly, all the piglets in medium, Δ658-777, or Δ56-inoculated groups survived and remained healthy. Piglets inoculated with wt HP-PRRSV HV had significantly lower average weight gain than piglets inoculated with medium (**Figure 9C**). Piglets inoculated with Δ658-777 or Δ56 mutant virus and the survived-piglets inoculated with Δ500-596 got about 4 kg weight at the end of the experiment, which was comparable to the increased weight of the control piglets.

Virus loads in serum samples were analyzed. As shown in **Figure 9D**, piglets infected with the mutant virus Δ500-596, Δ658-777, or Δ56 had significantly lower viral loads (~ 1-2 log lower) than piglets inoculated with wt HP-PRRSV. At the end of the experiment, viruses were almost undetectable in serum samples from Δ500-596-, Δ658-777-, or Δ56- infected piglets.

We also analyzed COX-2 expression in brain tissues (**Figure 9E**) and PGE2 production in cerebrospinal fluids (**Figure 9F**) at day 6 post-inoculation. COX-2 and PGE2 productions were significantly up-regulated in brains from piglets infected with wt HP-PRRSV compared to mock-infected piglets. And the mutant viruses Δ500-596, Δ658-777, and Δ56 induced significantly lower levels of COX-2 and PGE2 productions compared to wt HP-PRRSV.

Viral loads in lungs and brains from the euthanized pigs were analyzed by quantitative RT-PCR. The results showed that piglets infected with mutant virus Δ500-596, Δ658-777, or Δ56 had remarkably lower viral loads compared to the HV-infected piglets (**Figure 9G**). Histopathological examination revealed severe histopathologic damages in wt HP-PRRSV-infected piglets, including severe proliferative interstitial pneumonia in lungs, severe degrees of vasculitis in the brain, and thymic lesions of cortical involution, a poor demarcation between the cortical and medullary zones (**Figure 9H**). Piglets infected with Δ500-596 displayed alleviated histopathologic changes compared to piglets infected with wt HP-PRRSV, and only some slight microscopic lesions in lungs were observed. However, no histopathologic changes were observed in Δ658-777 or Δ56-infected piglets. These results indicate that mutant viruses Δ500-596, Δ658-777, and Δ56 cause less severe or no histopathologic lesions.

Taken together, these data suggest that NSP2 is important for HP-PRRSV to induce high fever in pigs.

## Discussion

In 2006, an atypical PRRS caused by HP-PRRSV was reported in China, which was identified with manifested neurological symptoms and severe pathological changes compared to the traditional PRRS (4). The most typical symptom was high fever (40-42°C). Here, we revealed that HP-PRRSV up-regulated COX-2 and PGE2 production in microglia *via* the activation of MEK1-ERK1/2-C/EBPβ signaling pathway. Of the viral proteins, NSP2 was demonstrated to induce COX-2 and PGE2 production through MEK1-ERK1/2-C/EBPβ pathways and played an important role in HP-PRRSV-triggered high fever. The key regions in NSP2 were aa 500-596 and aa 658-777. Importantly, the mutant HP-PRRS viruses with deletion of residues 500-596 and/or 658-777 in NSP2 were less virulent and failed to induce high fever responses *in vivo*.

Fever response, which is a highly preserved phylogenetic trait of acute inflammatory activation, is a hallmark of the host response to infections by microbial and viral pathogens (33). It has been reported that fever is strongly associated with significant morbidity and higher mortality (34, 35). For instance, in a swine model of acute myocardial infarction, elevated body temperature significantly increases infarct size (36). As to infection, it has been hypothesized that fever induces collateral tissue damages resulted from microbial killing mechanisms (37). Our previous study suggests that there is a negative correlation between high body temperature and the survival time for HP-PRRSV-infected piglets (5). And high fever is also related to virulence (38). Thus, it is necessary to explore the mechanisms on how HP-PRRSV triggers high fever in pigs.

Prostaglandins, especially PGE2, are demonstrated as the key mediators of the febrile response (10). Our previous report reveals that PGE2 is induced in PAMs by PRRSV infection, and increased PGE2 is observed in serum samples from HP-PRRSV-infected pigs (5). However, a previous report shows that even though high levels of PGE2 and PGE2 metabolites are recorded in plasma, there is no fever and no elevated PGE2 levels in CSF (39). In accordance with this report, some studies have shown that PGE2 produced by brain cells is considered to be the major pyrogenic mediator of fever in many models (12, 14, 18, 40). Given the pivotal role played by brain cell-derived PGE2 in the induction of fever, and the fact that PRRSV infects microglia (20), we used cultured primary porcine microglia to investigate how HP-PRRSV might induce high fever.

Generally, it is considered that COX-2 is the main regulator of PGE2 production. For example, the strong inflammation-induced expression of COX-2 in brain endothelial cells is crucial to febrile response (6). Mice deficient in COX-2 gene fail to develop fever in response to LPS challenge (24). Here, we found that the ability for HP-PRRSV to induce PGE2 production in microglia was significantly suppressed by COX-2 specific inhibitor celecoxib, suggesting that HP-PRRSV-infected PGE2 up-regulation is regulated by COX-2. We also found that COX-2 was up-regulated in HP-PRRSV-infected microglia and the brain tissues from HP-PRRSV-infected piglets. COX-2 is the rate-limiting enzyme in the PGE2 synthesis cascade, which can be induced upon cell activation and stimulation by pathophysiological stimuli. For example, poly(I:C), LPS, and viruses such as Japanese Encephalitis Virus and Chandipura Virus, can induce COX-2 expression in microglia (41–44). Interestingly, the COX-2/PGE2 signaling cascade is highly modulated by various viral infections, including hepatitis C virus, enterovirus71, cytomegalovirus, hepatitis B virus, and dengue virus (45–49). In addition, we also found that COX-1 inhibitor sc-560 decreased PGE2 secretion induced by HP-PRRSV, and COX-1 was also up-regulated by HP-PRRSV infection. However, studies have shown that COX-1 is dispensable for fever and is not strongly induced in brain during inflammation (50, 51). Interestingly, our previous study shows that PRRSV-induced PGE2 production in PAMs is mainly regulated by COX-1, but not COX-2 (5). Whether COX-1 plays an important role in PGE2 production induced by HP-PRRSV in microglia needs to be further investigated.

We also demonstrated that MEK1-ERK1/2-C/EBPβ signaling pathways were responsible for the augmentation of COX-2 and PGE2 in microglia infected with HP-PRRSV. The MAPK pathways are controlled by sequential activation of specific kinases. Mammalian MAPK superfamily, including ERK, p38 MAPK, and JNK, are regulated *via* Tyr/Thr phosphorylation by the upstream MAPK kinases MEK, MKK3/6, and MKK4/7, respectively (52). In our study, we observed that only the high specific MEK1 inhibitor, rather than other MAPK inhibitors, such as inhibitors for p38 MAPK and JNK, inhibited COX-2 and PGE2 production induced by HP-PRRSV. Similarly, MEK-ERK1/2 signaling pathway is activated by phosphatidylserine liposome in microglia to induce PGE2 production (49). There are many transcriptional factors downstream of MEK-ERK1/2 signaling pathways, including NF-κB, CREB, c-Fos, c-Jun, and C/EBPβ (52). Studies have reported that COX-2 is regulated by several transcription factors. For example, COX-2 production in macrophages induced by EMCV and dsRNA is sensitive to the inhibitor of NF-κB activation (53), and COX-2 and PGE2 production *in vitro* and *in vivo* in the inflamed central nervous system is regulated *via* C/EBPβ (26). Here, we found that the specific NF-κB inhibitor BAY11-7082 and specific AP-1 inhibitor SR11302 (data are not shown) had no effect on HP-PRRSV-induced COX-2 production and PGE2 secretion. Moreover, we only identified C/EBPβ as the crucial transcriptional factor for COX-2 induced by HP-PRRSV. The truncated COX-2 promoter (bases -177/+9) containing C/EBPβ binding motif (TTACGCAA) responded to PRRSV stimulation. However deletion of this C/EBPβ binding site significantly impaired COX-2 promoter activation by HP-PRRSV, confirming the importance of C/EBPβ in COX-2 production.

We screened HP-PRRSV proteins and found that NSP2 up-regulated COX-2 production through activating MEK1-ERK1/2-C/EBPβ signaling pathway. We then showed that NSP2 interacted with 14-3-3ζ to activate the signaling pathway. A previous study has investigated the interactome of NSP2, showing that 14-3-3 acts as an adaptor for NSP2 (28). 14-3-3 is a highly conserved and ubiquitously expressed protein family. It is reported that the interaction of 14-3-3 proteins with Raf-1 promotes Raf-1 conformations, which are catalytically competent to activate MEK-ERK signaling (54, 55). Additionally, we also found that transfection of NSP4 induced COX-2 production in 3D4/21 cells. However, whether and/or how NSP4 is involved in COX-2 induction and/or fever response need to be investigated in the future.

NSP2 is a multi-functional replicase subunit with different functional domains (56). A previous study suggests that the PL2 domain of NSP2 is responsible for interrupting NF-κB signaling in regulating interferon induction (57). And the HV domain of NSP2 can bind multiple cellular polypeptides (28). Using serial deletion mutants of HP-PRRSV NSP2 protein (NSP2 (161-1196) without PL2 domain, and NSP2 (846-1196), NSP2 (1031-1196), and NSP2 (Δ161-845) without HV domain), we showed that the HV domain (but not the PL2 domain) of NSP2 was responsible for COX-2 up-regulation. We further showed that residues 500-596 and 658-777 in NSP2 were essential for NSP2 to induce COX-2 expression. Indeed, the interaction between NSP2 and 14-3-3ζ was also abrogated by the deletion of residues 500-596 and 658-777. NSP2 structure is complex and not all of the domains within it are well defined. It is also possible that deletions of aa 500-596 and 658-777 have negatively impacted the overall protein’s folding, stability, and functionality. Structural information about NSP2 should be constructed in the future to unravel the reason why the residues 500-596 and 658-777 of NSP2 are the important regions for COX-2 induction.

We also assessed the ability of mutant HP-PRRS viruses, including Δ500-596, Δ658-777, and Δ56 to induce fever *in vivo*. Mutant HP-PRRSV-infected piglets had lower body temperature, indicating that residues 500-596 and 658-777 in NSP2 are important areas for HP-PRRSV to induce fever response. Thus, theoretically these areas might be associated with viral replication or virulence. Indeed, the recombined viruses had lower growth ability and weaker virulence compared to wt HP-PRRSV HV *in vivo*. This is inconsistent with a previous study, showing that varied genetic alterations in the PRRSV NSP2 gene, such as point mutations and nucleotide insertions or deletions, are related to the PRRSV virulence (58). For example, PRRSV strains, such as VR-2332, Em2007, MN184C, TJM-F92, and TJ, exhibit different levels of virulence in piglets (12, 59). The PRRSV strain Em2007, which contains a unique 68 amino acids deletion in NSP2, is a recombinant PRRSV variant of HP-PRRSV and the Chinese vaccine strain CH-1R (60). The MN184C strain with deletions at residues 324-434, residue 483, and residues 504-522 is a virulent isolate from the USA (61). The virulence of strains Em2007 and MN184C is weaker than that of HP-PRRSV HV strain, but higher than that of the prototype PRRSV-2 strain VR-2332 (29, 56, 62). TJM-F92 is a vaccine strain derived from the TJM strain of HP-PRRSV and contains deletions of residues 628-747, residue 481, and residues 532-560 (59). The discontinuous deletion of 30 amino acids (residue 481 and residues 532-560) in NSP2 was identified as novel virulence markers in the HP-PRRSV. However, a previous study has shown that this discontinuous deletion is not directly related to HP-PRRSV virulence (63). Interestingly, there are overlapping sequences between Em2007, TJM-F92, and our mutant viruses. We find that no matter in Em2007, TJM-F92, or our mutant viruses, deletions in NSP2 (except the discontinuous deletions of 30 amino acids) significantly reduce the virulence of HP-PRRSV, indicating that NSP2 might contribute to HP-PRRSV virulence. We cannot exclude the possibility that replication defects of the mutant viruses may contribute to the induction of lower body temperature. However, based on our *in-vivo* data, it seems that the higher COX2 and PGE2 induction is likely related to the feature of NSP2. At 2 days post-infection, pigs infected with HP-PRRSV already exhibit high temperatures, much higher than that of the pigs infected with the mutant viruses at days 10-13 post-infection. However, at days 10-13 post-infection, pigs infected with the mutant viruses have much higher virus loads than those in pigs infected with wt HP-PRRSV at day 2 post-infection.

**Figure 10:**
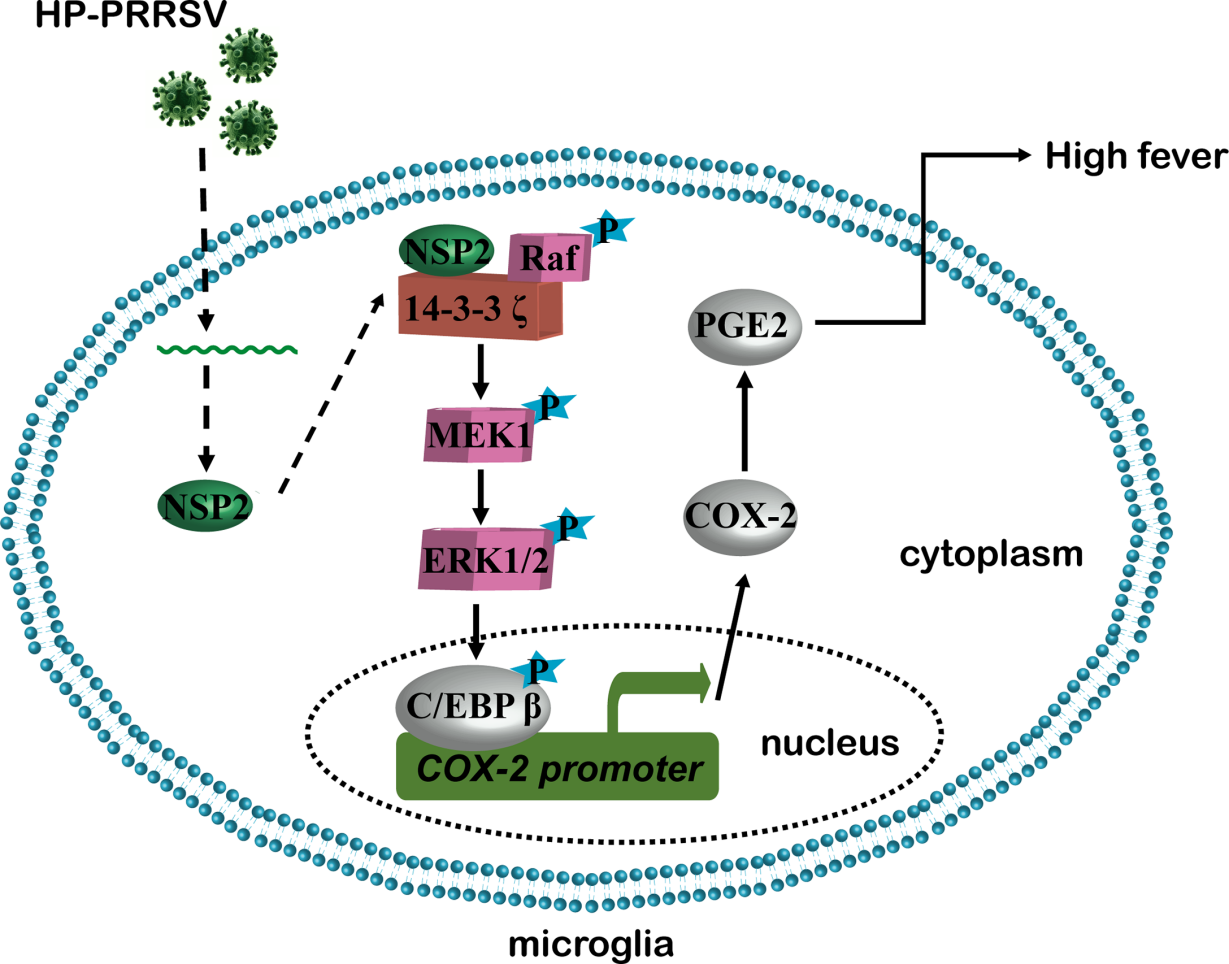
A working model of how HP-PRRSV triggers high fever.

In summary, our present study revealed the mechanism about how HP-PRRSV triggers high fever (**Figure 10**). We found that HP-PRRSV up-regulates COX-2 and PGE2 production by activating MEK1-ERK1/2-C/EBPβ pathways. Of the viral proteins, NSP2 was demonstrated to be important for HP-PRRSV to induce COX-2 and PGE2 production. Moreover, the residues 500-596 and 658-777 in NSP2 were identified as the essential regions. Importantly, we confirmed that the mutant viruses with the deletions of 500-596 and/or 658-777 in NSP2 could not induce high fever.

## Data Availability Statement

The original contributions presented in the study are included in the article/supplementary material. Further inquiries can be directed to the corresponding author.

## Ethics Statement

All animal trials in this study were performed according to the guidelines of the Beijing Laboratory Animal Welfare and Ethics of the Beijing Administration Committee of Laboratory Animals and were approved by the Beijing Association for Science and Technology (approval ID SYXK [Beijing] 2007-0023). The animal studies also complied with the China Agricultural University Institutional Animal Care and Use Committee guidelines (ID: SKLAB-B-2010-003) and were approved by the Animal Welfare Committee of China Agricultural University.

## Author Contributions

LD, HW, and W-hF conceived and designed experiments, analyzed data, and wrote the manuscript. LD, HW, FL, and ZW performed experiments. CW and JT provided critical reagents and scientific insights. All authors contributed to the article and approved the submitted version.

## Funding

This work was supported by the National Natural Science Foundation of China (Grant No. 31572516), the National Natural Science Foundation of China (Grant No. 31630076), and the National Key Research and Development Program of China (Grant No. 2017YFD0500601-1).

## Conflict of Interest

The authors declare that the research was conducted in the absence of any commercial or financial relationships that could be construed as a potential conflict of interest.
